# Exploring the potential of lightweight large language models for AI-based mental health counselling task: a novel comparative study

**DOI:** 10.1038/s41598-025-05012-1

**Published:** 2025-07-02

**Authors:** Ritesh Maurya, Nikhil Rajput, M. G. Diviit, Satyajit Mahapatra, Manish Kumar Ojha

**Affiliations:** 1 Department of Computer Science and Engineering, Madan Mohan Malaviya University of Technology, Gorakhpur, 273010 India; 2 Department of Artificial Intelligence, Amity University, Noida, 201303 India; 3 Department of Information and Communication Technology, Manipal Institute of Technology, Manipal Academy of Higher Education, Manipal, 576104 India

**Keywords:** Large language model, Mental health, Counseling, Artificial intelligence, Fine-tuning, Psychology, Human behaviour, Health care

## Abstract

In recent years, Transformer-based large language models (LLMs) have significantly improved upon their text generation capability. Mental health is a serious concern that can be addressed using LLM-based automated mental health counselors. These systems can provide empathetic responses to individuals in need while considering the negative beliefs, stigma, and taboos associated with mental health issues. Considering the large size of these LLMs makes it difficult to deploy these automated counselors on low cost/resource devices such as edge devices. Therefore, the motivation of the present study to analyze the effectiveness of lightweight LLMs in the development of automated mental health counseling systems. In this study, lightweight open source LLMs such as Google’s T5_s_ (small variant), BART_B_ (base variant), FLAN-T5_s_ (small variant), and Microsoft’s GODEL_B_ (base variant) have been fine-tuned for automated mental health counseling task utilizing a diverse set of datasets publicly available online. The experimental results reveal that BART’s base variant outperformed the other models across all key metrics such as ROUGE-1, ROUGE-2, ROUGE-L, and BLEU with scores of 0.4727, 0.2665, 0.3554, and 25.3993 respectively. In comparison to other models, BART-base model generated empathetic, and emotionally supportive responses. These findings highlight the potential of lightweight LLMs (small size LLMs), in advancing the field of LLM-based mental health counseling solutions and underscore the need for exploration of lightweight LLMs for this mental health counseling use case. The code for this work is available at the following link: https://github.com/diviitmg03/Comparative-analysis-of-LLMs-.git.

## Introduction

Mental health crisis is a serious global challenge, according to the WHO report one out of eight individual is suffering from mental health related issues^1^. As per the ICMR report(2017), 14.3% of the total population is suffering from mental disorders^2^. India faces a severe shortage of mental health professionals, with the availability of 0.75 psychiatrists per 100,000 people, far below the WHO’s recommended ratio of 3 per 100,000^3^.

In recent years, large language models(LLMs) have been used for a a wide variety of natural language processing-related applications, including question-answering, text generation, language summarization, etc^4^. Considering the advancement of LLMs in different fields, the applications of LLMs in the field of mental health counseling are still unexplored. The large size of these LLM models poses significant challenges in their deployment on low resource computing devices such as edge devices. Therefore, present study is aimed at exploring the potential of open source lightweight LLMs in the development of automated mental health counseling systems.

Most of the automated chat bots developed earlier for mental health counseling were either rule-based systems or relying on conventional machine learning models for their development^5^. The chat bots developed using such methods were designed for domain specific issues such as depression, anxiety, suicide prevention or stress management. The response generated by the conventional AI-based conversational agents was based on pre-defined rules without considering the need of an individual seeking mental counseling help. The major limitation of these types of systems were lack of generalisation to wide variety of mental health issues.

With the advancement of Transformer-based LLM models in recent years, it has been made possible to understand, analyze and respond in a meaningful manner to different mental health issues using LLM-based automated chatbots^6^. These LLM-based chatbots solves the limitations of rule-based chatbots earlier used for solving diverse mental health issues. LLM model once fine-tuned on large dataset can be deployed for wide variety of mental health counseling related issues. However, considering the large size( billion number of parameters) of these LLMs prevents their deployment on edge devices thereby, limits their scalability. Considering the problem of limited scalability of these LLM-based systems due to their large size, the proposed work analyses the effectiveness of lightweight LLMs(parameter count in few millions) in the development of automated chat bots for metal health counseling related tasks.

The primary hypothesis of this study is: **How do different lightweight language models perform in terms of accuracy and contextual relevance when generating responses for mental health counseling tasks under diverse scenarios?** To investigate this hypothesis, different lightweight LLMs, including T5_s_^7^, BART_B_^8^, FLAN-T5_s_^9^, and GODEL_B_^10^ have been fine-tuned on mental health conversation dataset. The performance and the contextual relevance of responses generated by these models were analyzed. The dataset used for fine-tuning these models comprised publicly available mental health counseling related conversations between patients and experts, structured in form of questions and corresponding responses, curated from different online platforms. A comprehensive evaluation of these models was performed to check their efficacy in generating contextually relevant and accurate responses within different mental counseling scenarios . Key performance metrics such as ROUGE (Recall-Oriented Understudy for Gisting Evaluation), BLEU(Bilingual Evaluation Understudy) and perplexity were used to benchmark the performance of these models. This detailed analysis provided valuable insights into the suitability and effectiveness of these lightweight LLMs for mental health counseling applications.

## Related works

Some of the works related to the present study have been discussed in this section.

The recent advancement of LLMs in the field of natural language processing has opened new possibilities in healthcare, particularly in addressing the mental health crisis. Several studies have been conducted to explore the potential of artificial intelligence in mental health. In the work^11^, researchers used rule-based systems in developing chatbots to address mental health-related issues. These systems generate synthetic responses in contrast to therapeutically relevant responses which are necessary for mental health counseling related task. In other work, researchers reviewed the use of AI in addressing diverse challenges in the field of mental health such as disease prevention, diagnosis and treatment interventions^12^. The researchers also proposed a method that uses data from various digital footprints of an individual, such as their social media posts and data related to their use of smartphones, to analyze the pattern and predict the mental health status of an individual^13^. In other work researchers analyzed the successful integration of AI in the field of mental healthcare concerning issues need to be addressed like privacy, bias and diagnostic accuracy^14^. Researchers also analyzed the ethical issues and the risk factors involved in the development of such AI-enabled chat bots such as over-reliance on the generated response and lack of emotional intelligence in these systems limits their adaptability in real life use case^15^. Schyff et al.^16^ proposed a conservational AI-based metal health support chatbot named Leora which provide support to its users in case of mild symptoms of anxiety and depression. The response generated by these AI-based conversational agents were generalized in nature and did not provide personalized assistance. Rathnayaka et al.^17^ in their work proposed an AI and behavioral activation-based personalized assistance for recurrent emotional support catering to the need of individual seeking therapeutic response to mental health related queries.

Recent advancements in the field of natural language processing with the development of Transformer-based LLMs models have significantly transformed healthcare. Peng et al.^18^ developed a model by fine-tuning the GPT-3 architecture with up to 20 billion parameters for healthcare text generation and biomedical natural language processing task. Singhal et al.^19^ have developed Med-PALM 2 LLM model for medical question answering task. This model achieved score of 67.2% on the MedQA dataset. Yang et al.^20^ proposed an ensemble of LLMs for medical question answering. He et al.^21^ fine tuned BERT model on knowledge of different disease such as their signs, symptoms, diagnosis and treatment. Despite the significant advancement of LLMs in the field of healthcare their adoption in the field of mental healthcare is in nascent stage or still unexplored systematically. Challenges like data scarcity, low user engagement and high drop out rates prevent the deployment of these LLMs in the field of mental healthcar^22,23^. Yadav et al.^24^ compared the state-of-the-art LLM models with and without fine-tuning and found that the fine-tuned LLMs performed better with improved generalization for automatic generation of diagnostic summaries for mental state screening. Zheng et al.^25^ generated dataset named ExTES (ExTensible Emotional Support dialogue dataset) and fine-tuned LLaMA model for mental healthcare with emotional support.

LLMs like Med-PaLM 2 have been successfully deployed in mental health diagnosis task with 92.5% accuracy in correctly diagnosing the depressive disorder^26^ Similarly, PaLM 2 model when fine-tuned on medical domain data generates more comprehensive list of psychiatric diagnosis in comparison to the medical experts^27^. Some group of researchers have proposed a framework to examining the issues such as bias, stereotyping, privacy violation and exacerbating inequalities in LLMs^28^ In other work researchers have proposed sociocultural-technical approach to address the challenges like technical costs, literacy gaps, biases, and inequalities^29^.

Considering the large size (billion number of parameters) of these LLMs which makes it impractical to deploy these models on low resource edge device. Therefore, in this work open source lightweight(parameters in few millions) LLMs have been explored for mental health counseling related task with special focus on generating empathetic, coherent, and contextually relevant response.

## Methodology

**Fig. 1:**
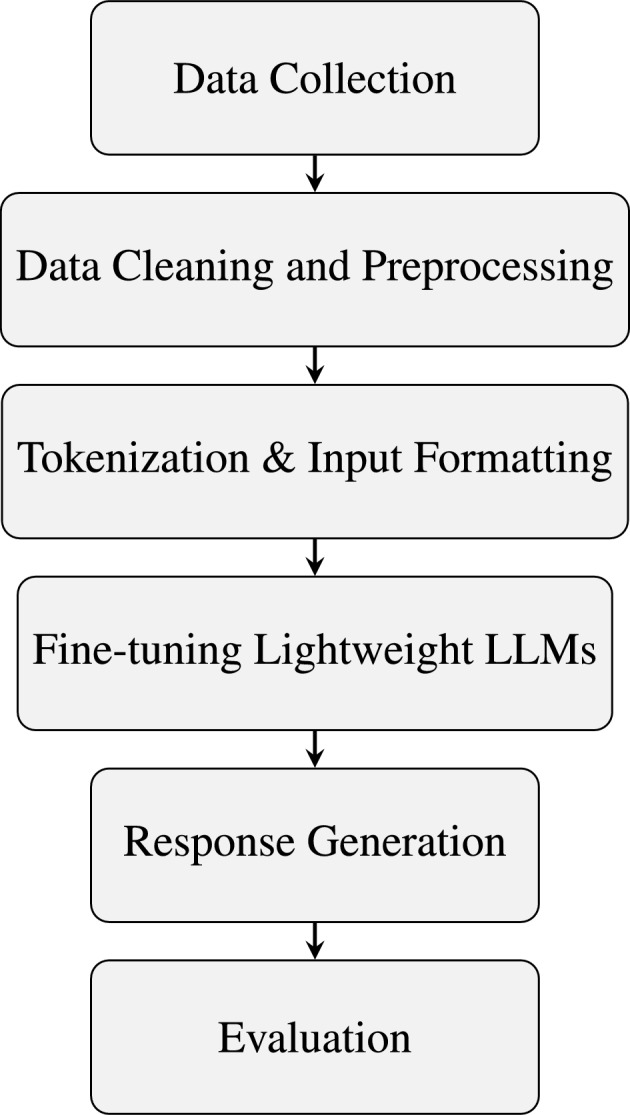
Overall methodology framework used in this study for fine-tuning and evaluating lightweight LLMs in mental health counseling.

The proposed method evaluates the effectiveness of the four four open-source lightweight LLMs (T5-small, FLAN-T5-small, BART-base, and GODEL-base) fine-tuned on curated mental health counseling conversation datasets. The proposed method involves steps like data preprocessing, tokenization, model fine-tuning, and evaluation using different metrics (ROUGE, BLEU, Perplexity), along with preliminary human feedback. The general flow of the proposed methodology is shown in Fig. 1. Detailed pictorial representation of the proposed methodology has been shown in Fig. 3. The following subsections provide a detailed explanation of the sub-processes involved in the proposed methodology.

### Data collection

**Fig. 2:**
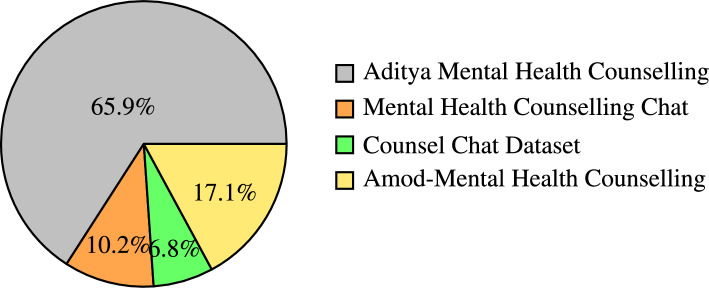
Distribution of Mental Health Datasets.

The datasets used in this work were curated from diverse online sources such as HuggingFace and GitHub. The collected data was organised in standardised format in form of question-response pairs, suitable for training of lightweight LLMs used in this study for the comparison purpose. The following datasets were used in this study for fine-tuning the LLMs: Aditya Mental Health Counselling Dataset^30^, Mental Health Counselling Chat^31^, Counsel Chat Dataset^32^ and Amod-Mental Health Counselling Conversations^33^. Diagramatic representation of the distribution of data among these datasets has been shown in Fig. 2.

Table 1 provides detailed information such as source, size and several other details about all four datasets used in this study for fine-tuning lightweight LLMs. The size of the combined datasets obtained after combining the instances of conversation from all four datasets into a single dataset was 20,500. Each instance consists of question and corresponding answer in form of question-answer pair in English language. The combined dataset consists of mental health related conversation from wide variety of topics such as depression, anxiety, self-esteem, coping mechanism, stress management and relationship issues.

Each dataset provides unique scenarios, enabling the fine-tuned language models to capture the details of various mental health concerns and respond with contextually appropriate and empathetic dialogue. The diversity in topics and conversational styles within the datasets supports a comprehensive training, promoting the development of language models capable of addressing complex mental health issues.

**Table 1:** Summary of four mental health datasets used in our experiment.

Dataset bame	Source	Task	Size (K)	Description
Aditya Mental Health Counseling^30^	Hugging Face, GitHub	Q&A	13.5	Contains Q&A pairs focused on mental health concerns like anxiety and stress.
Mental Health Counseling Chat^31^	Hugging Face	Dialogue generation	2.1	Conversations on depression, anxiety, and emotional challenges for therapy models.
Counsel Chat Dataset^32^	Counsel Chat, Hugging Face	Question & Answering	1.4	Licensed counselors respond to user-submitted questions on mental health.
Amod-Mental Health Counseling^33^	Hugging Face	Conversation modeling	3.5	Features dialogues on depression, mood swings, and self-care.

### Data cleaning

The first step in data cleaning process involves deletion of useless columns and duplicate entries to avoid repetition of data. Text fields in the dataset were cleaned by removal of HTML tags, extra strings and system generated prompts. The dataset was further organized into dialogue pairs in form of question and response. All four datasets cleaned in such a manner were combined further to utilise them for fine-tuning different lightweight LLMs.

**Fig. 3:**
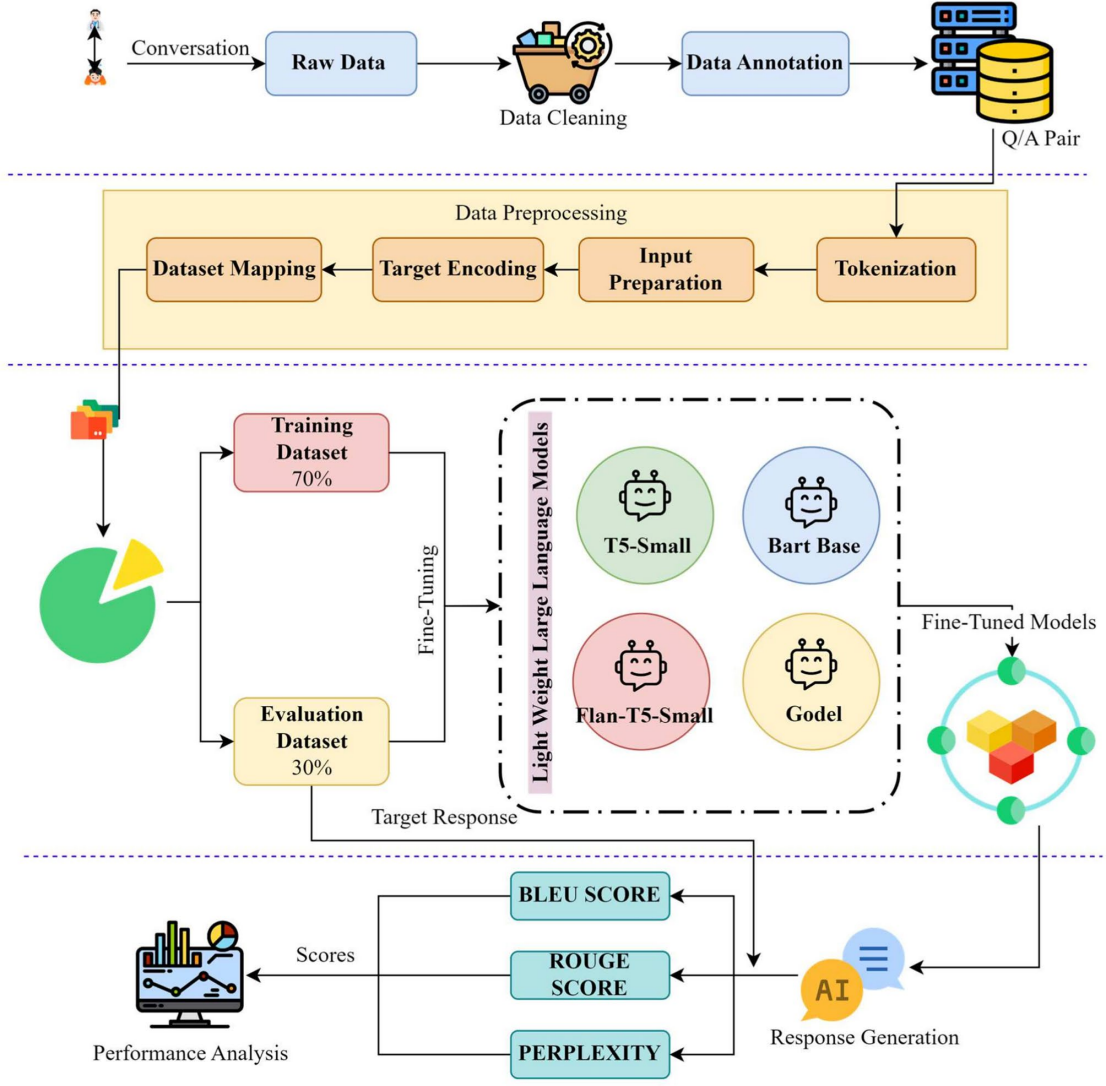
Flow graph for the propsoed methodology.

### Data pre-processing

The pre-processing pipeline starting from data preparation to tokenisation involved the following key steps:

**Input preparation and Tokenization:** Each question-answer pair in a the dataset was formatted as proper input form suitable for model’s input. In case of $\hbox {T5}_{S}$ and FLAN-T5_S_ models,each question in the dataset was pre-fixed with $``<question>''$ to contextualize the input. Contextualized input was then tokenised using ’T5Tokenizer’ from the Hugging Face Transformers library.It is based on SentencePiece tokenisation algorithm. Maximum sequence length was set to 512 to ensure that long conversations are truncated and padded as and when required to maintain the consistence in the length of input sequence.

**Target Encoding:** Transformers like T5 and BART expect separate tokenisation of responses corresponding to each question to generate the target sequences. Tokenisation on responses were applied using the same tokeniser as used in case of input questions. Maximum sequence length was set to 512 in case of target response generation. The encoded responses were assigned *“labels”* for supervised learning so that generated response and ground truth response can be compared.

**Dataset Mapping:** Dataset mapping was applied to both training as well as validation sets to ensures batch-wise tokenisation of question-answer pairs for efficent processing. This mapping ensures proper structuring of all input sequences for further processing by the LLMs.

### Selection and fine-tuning lightweight-language models

Different lightweight Sequence-to-sequence LLMs having a few million of parameters were selected and fine-tuned for mental health counseling conversation task. A detailed description of the selected lightweight-LLMs used in this study for the comparison purpose has been presented as follows:

**T5(Text-to-Text Transfer Transformer):** A prominent Seq2Seq model developed by Google with just only 60 million parameters makes it a suitable to be assessed for the present task of mental health counseling. It is based on text-to-text framework, takes text as an input and generates text thereon which makes in suitable for the present counseling conversation task. This model is efficient for wide variety of text generation tasks such as document summarization, question-answering and many more.In this work the ’small’ variant of T5 model has been used for the fine-tuning purpose^7^.

**BART(Bidirectional and Auto-Regressive Transformers):** BART model developed by Facebook takes inspiration from bidirectional text understanding capabilities of BERT (Bidirectional Encoder Representations from Transformers)-based encoder model and auto-regressive capabilities of GPT(Generative pre-trained transformer)-based decoder model. It is pre-trained on text corrupted through some noise function and it is good at text generation tasks like summarization, translation, question answering and text classification. In this work, the ’base’ variant of the BART model has been chosen considering its seq2seq framework with limited number (139 million) of parameters which makes it suitable for fine-tuning it on mental health counseling conversation task^34^.

**FLAN-T5:** FLAN is a family of improved transformers, in contrast to T5, FLAT-T5 has been instruction-tuned and improves the performance over T5 for diverse range of text generation tasks such as summarization, text generation, reasoning and question answering. FLAT-T5 is found to be more effective in zero-shot, few-shot and chain-of-thought reasoning.In this work FLAN-T5’s small size variant with 80 million parameters is used for fine-tuning on mental health counseling dataset^9^.

**GODEL(Grounded Open Dialogue Language Model):** Developed by Microsoft Research, it is an open-source pre-trained language model which is a successor of Microsoft’s DialoGPT model and it was primarily developed for goal-oriented dialogue. This model is based on encoder-decoder architecture and it is well trained for generating response conditioned on some external text^10^. The reason behind choosing this model is based on its suitability for dialogue generation task with only 220 million parameters.

The detailed description about each of these models has been provided in Table 2. These models were fine-tuned on the pre-processed combined dataset using the hyperparameters presented in experimental results section. The detailed description of these hyper-parameters has been provided in the next section.

**Table 2:** Comparison of different language models used in the study.

Model	Size (in million)	Open source	Architecture type
BART$_B$^8^	139	Yes	Transformer (Bidirectional Encoder-Autoregressive Deoder)
FLAN-T5$_S$^9^	80	Yes	Transformer (Encoder-Decoder)
T5$_S$^7^	60	Yes	Transformer (Encoder-Decoder)
GODEL$_B$^10^	220	Yes	Transformer (Pretrained Language Model)

### Response generation and evaluation of fine-tuned LLMs

The combined dataset was split into train and test set: 70% of the combined dataset was used for training whereas 30% of it was used for testing purpose. The questions in the test set were given as an input to these fine-tuned lightweight LLMs and the response generated by these LLMs were recorded and compared with the actual responses present in the dataset. Several quantitative metrics such as ROUGE^35^, BLEU^36^, and perplexity^37^, have been used to evaluate the effectiveness of the response generated by these fine-tuned models. These scores helps in evaluating the quality, relevance, and reliability of the response generated by these models.

ROUGE score evaluates the overlap between generated text and targeted response by evaluating recall across n-grams. The evaluation of ROUGE score ensures that the response generated by these models are aligned with targeted response.

BLEU (Bilingual Evaluation Understudy) score measures the precision of n-gram overlap between the generated response and the target response. High BLEU scores can contribute in generating more fluent and relevant responses, which may help in the improving the user engagement in mental health counseling conversations.

Perplexity score quantifies the amount of uncertainty or model’s confidence in generated response. Lower perplexity score denotes higher confidence in the prediction made by the model and vice versa for the higher perplexity score. A low perplexity score ensures the consistent and coherent interactions during mental health counseling conversation.

Using the ROUGE and BLEU score, informativeness and lexical similarity of the generated responses have been measured, whereas the lower perplexity score indicates a more coherent and a well-trained fine-tuned model. Altogether, these metrics provide a comprehensive evaluation framework, assessing the text similarity and the predictability of the fine-tuned LLMs; however, empathy and contextual relevance of the generated responses have also been assessed by the human evaluator.

The proposed methodology has been outlined in Fig. 3, starting from data collection, pre-processing , splitting, fine-tuning and evaluation of response generated by lightweight LLMs for mental health counseling conversation. 70% of the combined dataset was used for training whereas 30% of the dataset was used for testing purpose. The responses generated by these models were compared against the target response and different performance metrics were evaluated. The generated responses were evaluated against ROUGE, BLEU and Perplexity scores, in addition to that the generated responses were also evaluated against contextual coherence and emphatic reply generated by these fine-tuned LLMs under different counseling scenarios using human evaluator.

## Experimental results and discussions

### Experiment setup and performance metrics

All experiments were performed with a NVIDIA T4 GPU with 16GB of RAM. Test dataset consists of approximately 4,000 mental health counseling dialogues, used for testing the trained models. The evaluation was performed by comparing the model-generated responses against the reference responses.

### Hyperparameters used

The hyperparameters used in this work for fine-tuning the LLMs are summarized in Table 3. Batch size was set to 8 and the learning rate was set to $5 \times 10^{-5}$. The fine-tuned models were trained for 50 epochs to ensure proper convergence without any sign of overfitting. Other hyperparamters like weight decay was set to 0.01 for regularization and mixed precision (fp16) was used for better efficiency and storage complexity.

**Table 3:** Hyperparameters used for evaluating the language models in this study.

Hyperparameter	Value
Batch size	8
Epochs	50
Learning rate	$5 \times 10^{-5}$
Weight decay	0.01
fp16	True
Max length	512
Padding	’max-length’
Truncation	’true’
Gradient accumulation steps	2

### Tokenisers

The details of the tokenisers and tokenisation approach used with the different fine-tuned LLM models used in this work are presented in Table 4

**Table 4:** Tokenisers and tokenisation approach.

Model	Tokenizer used	Tokenization approach
$\text {T5}_\text {S}$	T5Tokenizer	SentencePiece
$\text {FLAN-T5}_\text {S}$	T5Tokenizer	SentencePiece
$\text {BART}_\text {S}$	BartTokenizer	Byte-Pair Encoding (BPE)
$\text {GODEL}_\text {S}$	T5Tokenizer	SentencePiece

### Performance comparison

Different performance metrics used to compare the response generated by the fine-tuned models with the target response have been presented in Table 5. Different performance metrics such as ROUGE-1, ROUGE-2, ROUGE-L, and BLEU scores were used to compare the performance of the models. The comparison of the fine-tuned models on these parameters ensure that the responses generated by these models are contextually relevant and coherent.

Among all the models, **BART**_**B**_ model achieved highest score across all the metrics, achieving a ROUGE-1 score of **0.4727**, ROUGE-2 of **0.2665**, ROUGE-L of **0.3554** and BLEU score of **25.3993**. These metrics ensures that the responses generated by the BART_B_ model are contextually relevant as well as coherent. **GODEL**_**B**_ model achieved moderate performance with a BLEU score of **6.6183** and reasonable ROUGE scores. The response generated by GODEL_B_ model as presented in Table 6 is found to be emphatic though it lacks specificity as observed in the response generate by the BART_B_ model. The performance of FLAN-T5_S_ and T5_S_ model was not found satisfactory as FLAN-T5_S_ model achieved a ROUGE-1 score of **0.2632** and BLEU score of **3.0431**, whereas in case of T5_S_ model ROUGE-1 score of **0.2585** and BLEU score of **3.0649** was achieved which suggest the limitations of T5_S_ and FLAN-T5_S_ model in capturing the . linguistic subtleties and psychological nuances necessary for the generation of comprehensive counseling responses.

**Table 5:** Performance metrics comparison.

Model	ROUGE-1	ROUGE-2	ROUGE-L	BLEU
T5_S_	0.2585	0.0877	0.1914	3.0649
Flan-T5_S_	0.2632	0.0954	0.1990	3.0431
$\text {BART}_\text {B}$	**0.4727**	**0.2665**	**0.3554**	**25.3993**
GODEL_B_	0.3350	0.1324	0.2328	6.6183

To analyse the linguistic coherence and psychological relevance of the response generated by these fine-tuned models, a comparison of the perplexity score obtained by these models when a common question is posed *“Can you help me with understanding how to deal with anxiety?”* to these models as presented in Table 6 .

**T5**_B_ model achieved lowest perplexity score of **1.78**, representing the fluency and coherence of the generated text. However, the response generated by T5_B_ model against the common question was found to be repetitive and it also lack practical utility, undermining of the use of T5_B_ model in real life mental health counseling applications.

GODEL_B_ achieved a perplexity score of **3.40** while the response generated by the model was found to be in empathetic tone, supporting anxiety but lack actionable detail. **FLAN-T5-small**, with a higher perplexity score of **6.20**, generated concise but less fluent and psychologically irrelevant response.

Though the BART_B_ model achieved a perplexity score of **3.75**, however, considering the fluency, physiological depth, actionable advice and well-framed structure of the generated response, BART_B_ model among other models provide most coherent response making it a suitable model for the implementation of LLM-based mental health counseling. In contrast, considering the low perplexity score of T5_B_ model suggest that it prioritizes fluency over the depth of a generated response whereas in case of GODEL_B_ and FLAN-T5_S_ model generated response focuses more on empathy than providing actionable advice which is necessary for therapeutic counseling use case.

**Table 6:** Responses generated by different models and a comparison of their perplexity scores for the common question, “Can you help me understand how to deal with anxiety?”.

Model	Generated text (Summary)	Perplexity	Analysis
GODEL_B_	“Anxiety can be a difficult thing to deal with... Let’s work together to identify the triggers...”	3.40	Empathetic response generation or human-like anxiety support.
BART_B_	“This question is a great one! Anxiety is a treatable condition... Talk to a therapist... Practice deep breathing exercises...”	3.75	Structured actionable suggestions or comprehensive practical advice.
Flan-T5_s_	“Anxiety can be a difficult emotion to manage... We can work together to develop coping strategies...”	6.20	Concise coping strategies or less detailed, higher perplexity.
T5_s_	“Can you help me with understanding how to deal with anxiety? Can you help me with understanding how to deal with anxiety?”	**1.78**	Repetitive and nonsensical responses.

### Analysis of real-time response generation

To analyse the effectiveness of the real-time response generation capabilities of the fine-tuned models used in this study, the response generated by these models were assessed for their contextual coherence, relevance, empathy in addressing the mental health related queries. For this purpose, two contextually different questions were posed to each of these fine-tuned models and the responses generated by these models were presented in Tables 7 and 8.

### Response to question 1: crying during therapy

Analysis of the response generated by these fine-tuned models as presented in Table 7, for the question *“Is it normal for people to cry during therapy, or is it just me?”* has been made in this subsection. The response generated by the BART_B_ model was found to be coherent and empathetic, addressing emotional support and providing reassurance in a compassionate tone. The response generated by the FLAT-T5_s_ model was also empathetic and relevant; however, occasional repetitiveness in the generated response diminishes its fluency. In contrast, the responses generated by the GODEL_B_ and T5_S_ models suffer from redundancy and contradictions, thereby diminishing the reliability of these models in generating contextually coherent and empathetic responses.

### Response to question 2: solutions for panic attacks

Analysis of the response generated by these fine-tuned models as presented in Table 8, for the question *“Give me solution for my panic attack.”* has been made in this subsection. The response generated by the BART_B_ model was found to be structured and practical, suggesting suitable coping mechanism and actionable advice useful in case of therapeutic context. FLAN-T5_S_ provides response to the question in a supportive tone but it lacks detailed actionable advice, therefore, the response generated by the FLAN-T5_S_ small was found to be less effective than the response generated by the BART_B_ model. GODEL model provides a brief and generic response, without any actionable advice. The response generated by the T5_S_ model is found to be contextually irrelevant with contradictory statements.

**Table 7:** Responses from different models to the question: “Is it normal for people to cry during therapy, or is it just me?”.

Question: Is it normal for people to cry during therapy, or is it just me?	
**BART**_**B:**_ Yes, definitely crying is normal during therapy. Anyone in the midst of new awareness of how they’ve been hurt, or feel despair or great sadness from burdensome life situations, naturally will feel tears come up. Therapists are familiar with people crying during a session and will know how to be present for you while you sort through your painful emotions.	
*GODEL*_*B:*_ Yes, it is absolutely normal to cry in therapy. Therapy is a place where you can be vulnerable and you can be vulnerable. It is a place where you can be vulnerable and you can be vulnerable. (Repeated multiple times).	
FLAN-T5_s:_Yes, it’s normal to cry during therapy. Therapy is a place where you are able to express your feelings in a safe and supportive environment. It’s also a place where you can express your feelings in a safe and supportive environment.	
T5_s:_ It’s normal to cry during therapy, but it’s not normal to cry during therapy. It’s not normal to cry during therapy, but it’s not normal to cry during therapy.

**Table 8:** Responses from different models to the question: “Give me solution for my panic attack.”.

Question: Give me solution for my panic attack.	
**BART**_**B:**_ Panic attacks can be scary and overwhelming, but there are a few things you can do to help yourself cope with them. One thing I would suggest is to find a therapist who specializes in panic attacks. They can help you learn healthy coping mechanisms to manage your panic attacks, such as deep breathing exercises, mindfulness techniques, and cognitive restructuring. You can also contact me via my website: http://psychcentral.com/lib/panic-anxiety-disorder/basics/what-is-the-best-case-right-now/?page=2	
*GODEL*_*B:*_ Panic attacks can be scary and overwhelming. Let’s explore what might be causing your panic attacks and see if we can come up with a solution that works for you.	
FLAN-T5_s:_ Panic attacks can be very distressing, but it’s important to remember that you are not alone. Let’s work together to identify the triggers for your panic attacks and develop coping strategies to manage them. We can also explore any underlying issues that may be contributing to your panic attacks.	
T5_s:_ I’m sorry to hear about your panic attacks. I’m sorry to hear about your panic attacks. I’m sorry to hear about your panic attacks.	

Comparing the responses generated by fine-tuned lightweight LLMs for Question 1 and Question 2 as presented in Tables 7 and 8 reveals that the BART_B_ model generated detailed, actionable and emphatic response aligning with the principle of mental health counseling. FLAT-T5_S_ model generated response with emphatic tone but lacks in depth required to be utilised in practical use cases. The responses generated by the GODEL and T5_S_ model lacks detail and suffered with redundancy and contradiction.

Table 9 presents a comparative analysis of language models used in this study on the basis of their memory requirement and inference speed. Despite the significant difference between the memory requirement of these language models, these models can easily be deployed on the mobile devices considering their small memory requirement and fast inference speed.

**Table 9:** Comparison of lightweight LLMs in terms of parameter count, model size, and inference speed.

Model	Parameters (in Millions)	Size (MB)	Inference speed (ms/input)
${\hbox {T5}}_B$	60M	$\sim$140	$\sim$45
FLAN-T5$_S$	80M	$\sim$160	$\sim$50
${\hbox {BART}}_B$	139M	$\sim$480	$\sim$70
${\hbox {GODEL}}_B$	220M	$\sim$500	$\sim$75

All the models evaluated in this study are lightweight enough to be deployed on low cost GPUs or even on mobile System-on-Chip (SoC) devices. The low memory footprint and fast inference make these models well suited in real world scenarios. Although in previous studies^26,27^ LLMs like GPT-3 or LLaMA have shown decent performance on mental health related conversation tasks. Considering the high computational demands of these LLMs in terms of memory and compute, their deployment at end devices is still not plausible.

These findings highlights the potential of BART_B_ model for its application in the implementation of mental healthcare support system.

## Limitations and future work

Though the present study provides a valuable insight into the application of lightweight LLMs in the implementation of automated mental health question-answering task, the following limitations of the proposed study should also be acknowledge: The datasets utilised in this study though collected from diverse online resources the verifiability of collected data limits its use in diverse mental healthcare contexts. Considering the focus of the present study on small LLMs with limited parameters, the large or medium size LLMs with more training data may improve the performance in real-life use cases of mental healthcare.

While automatic metrics such as ROUGE, BLEU and Perplexity scores have been used to evaluate the response generated by the language models used in this study, a preliminary human assessment by single non-expert evaluator have also been made to assess the coherence and empathy of generated responses. However, we acknowledge the limitations of this informal evaluation. In future work, we plan to involve mental health counselors and users to develop human-in-loop-based framework providing domain-specific feedback and also providing more rigorous assessment of the emotional and therapeutic effectiveness of the model-generated responses.

## Ethical considerations

Deployment of language models for mental health counseling raises several ethical concerns. Some of the issues are the generation of inappropriate or misleading responses and hallucinations, which could be harmful if interpreted as professional advice. Therefore, users of AI-based conversational systems should be informed that all the advices are AI generated, and the responses generated by the language models should not be taken seriously without the advice of an expert physiologist.

Another ethical consideration is related with the data privacy related to the sensitive personal information. Moreover, inherent biases in training data might lead to biased responses requiring future efforts in bias detection and mitigation.

Responsible deployment of these systems should follow a human-in-the-loop framework and must involve mental health professionals to assure safety and fairness,in practical use.

## Conclusion

This study investigated the potential of lightweight Large Language Models (LLMs) for AI-driven mental health counseling, focusing on four models–Microsoft-Godel, BART-base, T5-small, and FLAN-T5-small–fine-tuned on a curated dataset derived from diverse, high-quality mental health counseling resources. Among these models, BART-base consistently achieved the highest ROUGE-1, ROUGE-2, ROUGE-L, and BLEU scores, demonstrating its superior capability to generate coherent, contextually relevant linguistically accurate responses. The responses generated by fine-tuned lightweight LLMs were also analyzed based on empathy and emotional supportiveness. These results underscore the viability of lightweight LLMs like BART-base for mental health applications, even in scenarios requiring nuanced and emotionally supportive communication.

Furthermore, the study highlights the critical role of a curated and diverse dataset in fine-tuning LLMs for specialized tasks such as mental health counseling. The dataset not only enhanced model performance but also served as a valuable foundation for fine-tuning LLMs to this sensitive domain. With further improvements in data quality and validation, it could enable the development of robust AI-powered mental health counseling tools.

Overall, this research demonstrates the potential of lightweight, accessible LLMs to contribute to mental health interventions, offering scalable and cost-effective solutions. Future research should explore refining these models for broader generalizability, integrating ethical frameworks to ensure safe deployment, and addressing the challenges of real-world implementation to advance the field of AI-driven mental health counseling support system. Future research should also focus on curating and validating a more diversified and high-quality dataset to enhance the robustness and effectiveness of these models.

## Data Availability

The datasets used in this study have been collected from the following sources.Mental Health Counselling Dataset on Hugging Face, Chat Data on GitHub, dataset for counseling dialogues and Mental Health Counseling Conversations on Hugging Face.

## References

[1] Who mental health report. https://www.who.int/teams/mental-health-and-substance-use/world-mental-health-report.

[2] Collaborators, I.S.-L.D.B.I.M.D. The burden of mental disorders across the states of India: The global burden of disease study 1990–2017. *Lancet Psychiatry* **7**, 148–161. 10.1016/S2215-0366(19)30475-4 (2020). 31879245 10.1016/S2215-0366(19)30475-4 PMC7029418

[3] National mental health survey. https://science.thewire.in/health/the-case-to-expand-psychiatric-education-for-mbbs-students/.

[4] Bubeck, S. *et al.* Sparks of artificial general intelligence: Early experiments with gpt-4. *Preprint at arXiv* (2023). arXiv:2303.12712.

[5] The rise of ai in mental health care. https://trendsresearch.org/insight/smart-therapy-solutions-the-rise-of-ai-in-mental-health-care/#:~:text=In%20the%20realm%20of%20mental,enhancing%20treatment%20accessibility%20and%20effectiveness.

[6] Denecke, K., Abd-Alrazaq, A. & Househ, M. *Artificial intelligence for chatbots in mental health: Opportunities and challenges.*10.1007/978-3-030-67303-1_10 (2021).

[7] Google. T5 small. https://huggingface.co/google-t5/t5-small (2023).

[8] Facebook. Bart base. https://huggingface.co/facebook/bart-base (2023).

[9] Google. Flan-t5 small. https://huggingface.co/google/flan-t5-small (2023).

[10] Microsoft. Godel v1.1 large seq2seq. https://huggingface.co/microsoft/GODEL-v1_1-large-seq2seq (2023).

[11] D’Alfonso, S. Ai in mental health. *Curr. Opin. Psychol.* **36**, 112–117. 10.1016/j.copsyc.2020.04.005 (2020). 32604065 10.1016/j.copsyc.2020.04.005

[12] Feng, X., Hu, M. & Guo, W. Application of artificial intelligence in mental health and mental illnesses. In *Proceedings of the 3rd International Symposium on Artificial Intelligence for Medicine Sciences*, 506–511, 10.1145/3570773.3570834 (Association for Computing Machinery, New York, NY, USA, 2022).

[13] Darzi, P. Could artificial intelligence be a therapeutic for mental issues?. *Sci. Insights* **43**, 1111–1113. 10.15354/si.23.co132 (2023).

[14] Shimada, K. The role of artificial intelligence in mental health: A review. *Sci. Insights* **43**, 1119–1127. 10.15354/si.23.re820 (2023).

[15] Abd-Alrazaq, A. A. et al. An overview of the features of chatbots in mental health: A scoping review. *Int. J. Med. Inform.* **132**, 103978. 10.1016/j.ijmedinf.2019.103978 (2019). 31622850 10.1016/j.ijmedinf.2019.103978

[16] van der Schyff, E., Ridout, B., Amon, K., Forsyth, R. & Campbell, A. Providing self-led mental health support through an artificial intelligence-powered chat bot (leora) to meet the demand of mental health care. *J. Med. Internet Res.* **25**, e46448. 10.2196/46448 (2023). 37335608 10.2196/46448 PMC10337342

[17] Rathnayaka, P. et al. A mental health chatbot with cognitive skills for personalised behavioural activation and remote health monitoring. *Sensors*10.3390/s22103653 (2022). 35632061 10.3390/s22103653 PMC9148050

[18] Peng, C. et al. A study of generative large language model for medical research and healthcare. *npj Digital Med.* **6**, 210. 10.1038/s41746-023-00958-w (2023). 10.1038/s41746-023-00958-w PMC10654385 37973919

[19] Singhal, K. et al. Towards expert-level medical question answering with large language models (2023). arXiv:2305.09617. 10.1038/s41591-024-03423-7 PMC11922739 39779926

[20] Yang, H. et al. *One llm is not enough: Harnessing the power of ensemble learning for medical question answering.*10.1101/2023.12.21.23300380 (2023).

[21] He, Y., Zhu, Z., Zhang, Y., Chen, Q. & Caverlee, J. Infusing disease knowledge into bert for health question answering, medical inference and disease name recognition (2020). arXiv:2010.03746.

[22] Stade, E. C. et al. *Large language models could change the future of behavioral healthcare: a proposal for responsible development and evaluation*10.1038/s44184-024-00056-z (2024). 10.1038/s44184-024-00056-z PMC10987499 38609507

[23] Lai, T. et al. Supporting the demand on mental health services with ai-based conversational large language models (llms). *BioMedInformatics* **4**, 8–33. 10.3390/biomedinformatics4010002 (2024).

[24] Yadav, M., Sahu, N. K., Chaturvedi, M., Gupta, S. & Lone, H. R. Fine-tuning large language models for automated diagnostic screening summaries (2024). arXiv:2403.20145.

[25] Zheng, Z., Liao, L., Deng, Y. & Nie, L. Building emotional support chatbots in the era of llms (2023). arXiv:2308.11584.

[26] Galatzer-Levy, I. R., McDuff, D., Natarajan, V., Karthikesalingam, A. & Malgaroli, M. The capability of large language models to measure psychiatric functioning. arXiv preprint arXiv:2308.01834, 10.48550/arXiv.2308.01834 (2023).

[27] McDuff, D., Schaekermann, M., Tu, T. & et al. Towards accurate differential diagnosis with large language models. arXiv preprint arXiv:2312.00164, 10.48550/arXiv.2312.00164 (2023). 10.1038/s41586-025-08869-4 PMC12158753 40205049

[28] Solaiman, I. et al. Evaluating the social impact of generative ai systems in systems and society. arXiv preprint arXiv:2306.05949, 10.48550/arXiv.2306.05949 (2023).

[29] Malgaroli, M. et al. Large language models for the mental health community: Framework for translating code to care. *Lancet Dig. Health* **7**, e282–e285. 10.1016/S2589-7500(24)00034-6 (2024). 10.1016/S2589-7500(24)00255-3 PMC11949714 39779452

[30] Aditya. Mental health counselling dataset. https://huggingface.co/datasets/Aditya149/Mental_Health_Counselling_Dataset (2023).

[31] Bertagnolli, N. Counselchat dataset. https://github.com/nbertagnolli/counsel-chat/blob/master/data/counselchat-data.csv (2020).

[32] Bertagnolli, N. Counsel chat: A dataset for counseling dialogues. https://huggingface.co/datasets/nbertagnolli/counsel-chat (2023).

[33] Amod. Mental health counseling conversations. https://huggingface.co/datasets/Amod/mental_health_counseling_conversations (2023).

[34] Bart model overview. https://huggingface.co/docs/transformers/en/model_doc/bart.

[35] Face, H. Rouge metric (2023).

[36] GeeksforGeeks. Nlp - bleu score for evaluating neural machine translation in python. https://www.geeksforgeeks.org/nlp-bleu-score-for-evaluating-neural-machine-translation-python/ (2023).

[37] Face, H. Perplexity in transformers. https://huggingface.co/docs/transformers/en/perplexity (2023).

